# Metabolic syndrome and prostate cancer risk in a population-based case–control study in Montreal, Canada

**DOI:** 10.1186/s12889-015-2260-x

**Published:** 2015-09-18

**Authors:** Audrey Blanc-Lapierre, Andrea Spence, Pierre I. Karakiewicz, Armen Aprikian, Fred Saad, Marie-Élise Parent

**Affiliations:** Epidemiology and Biostatistics Unit, Institut national de la recherche scientifique-Institut Armand-Frappier, University of Quebec, 531 Boul. des Prairies, Laval, QC H7V 1B7 Canada; Cancer Prognostics and Health Outcomes Unit, University of Montreal Health Center, 1058, rue St-Denis, Montreal, QC H2X 3 J4 Canada; Department of Surgery, Division of Urology, Centre Hospitalier de l’Université de Montréal, 1058, rue St-Denis, Montreal, QC H2X 3 J4 Canada; Department of Surgery (Urology), McGill University Health Centre, 1650 Cedar Avenue, Montreal, QC H3G 1A4 Canada; Department of Social and Preventive Medicine, University of Montreal, 7101 Avenue du Parc, Montreal, QC Canada

**Keywords:** Metabolic syndrome, Prostate cancer, Case–control studies, Epidemiology, Risk factors

## Abstract

**Background:**

The role of metabolic syndrome (MetS) in prostate cancer risk is still debated. We investigated it in a large population-based case–control study.

**Methods:**

Cases were 1937 men with incident prostate cancer, aged ≤75 years, diagnosed across French hospitals in the Montreal area between 2005 and 2009. Concurrently, 1995 population controls from the same residential area and age distribution were randomly selected from electoral list of French-speaking men. Detailed lifestyle and medical histories, and anthropometric measures, were collected during in-person interviews. Prevalence of MetS components (type 2 diabetes, high blood pressure, dyslipidemia and abdominal obesity) was estimated at 2 years before diagnosis for cases/ interview for controls, and at ages 20, 40, 50 and 60. Logistic regression was used to estimate odds ratios (OR) and 95 % confidence intervals for the association between MetS and prostate cancer risk.

**Results:**

A history of MetS (≥3 components vs <3) was associated with a reduced risk of prostate cancer (OR = 0.70 [0.60, 0.82]) after considering potential confounders. The negative association was particularly pronounced with a young age (≤40 years) at MetS onset (OR = 0.38 [0.16-0.89]), did not vary according to prostate cancer aggressiveness, and was only partly explained by the presence of type 2 diabetes. A risk decrease was observed with the number of MetS components, suggesting a synergistic interaction of the components.

**Discussion:**

The observed negative association, consistent with results from other North American populations undergoing regular prostate cancer screening, underlines the importance of considering PSA-testing when studying the MetS-prostate cancer association.

**Conclusions:**

Findings from this study are consistent with an inverse association between MetS and prostate cancer risk.

## Background

Prostate cancer (PCa) is the most frequent non-skin cancer diagnosed in men in the western world [1]. The only established risk factors (age, family history of PCa and ancestry) are not modifiable [2]. Evidence from migration studies provide support for a role of environmental factors in PCa etiology [3]. Parallel increases in rates of PCa and metabolic disorders in North America suggest that factors associated with westernization, such as diet and physical activity, may be involved in PCa carcinogenicity [4, 5].

Metabolic syndrome (MetS), defined as a cluster of metabolic disorders associated with insulin resistance and visceral adiposity, was first used to identify subjects at increased risk of type 2 diabetes (T2D) and cardiovascular diseases. Different definitions of MetS have been proposed since 1998, varying from a glucocentric definition to an obesity-centric one, but all including glucose intolerance (high fasting glucose blood level), dyslipidemia (high triglycerides [TG] or low high-density lipoprotein cholesterol [HDL-C] blood levels), hypertension and abdominal obesity [6, 7]. MetS represents a growing public health concern given its high prevalence worldwide [8], especially in the United States where one third of the adult population is currently affected [4, 9].

MetS is suspected to influence the regulation of PCa growth and progression through various pathways, including the IGF-1 pathway stimulated by hyperinsulinemia, the sex steroid pathway (increased estradiol, decreased sex hormone-binding globulin and lower testosterone levels) and inflammation mediated by cytokines and hormones produced by adipocytes [10–13]. Although inter-related, MetS components affect PCa risk differently when considered separately. For instance, obesity assessed using the body mass index (BMI) is associated with an increased risk of high-grade PCa, but data remain insufficient regarding the specific role of abdominal fat [14–18]. Long-standing diabetes is associated with decreased incidence of PCa [19, 20]. Thus, metabolic disorders have to be considered together when evaluating their relation with PCa in order to provide useful guidelines for the management of PCa risk by physicians [21].

Recent investigations integrating multiple MetS components into a single condition have shown positive, negative or no relationship with PCa risk [10, 22, 23]. Studies have usually relied on MetS status at study baseline, precluding the evaluation of MetS timing in relation to PCa, which may be of importance for a disease with a long natural history such as PCa. We provide here new evidence for the association between MetS and PCa, using data from a large Canadian population-based case–control study.

## Methods

### Study population

The Prostate Cancer & Environment Study (PROtEuS), described previously [24–27], is a population-based case–control study conducted in Montreal, Canada, to assess the role of environmental factors in PCa risk. Eligible subjects were men, younger than 76 years of age at the time of diagnosis or selection, residents of the greater Montreal area, registered on Quebec’s permanent electoral list (continually updated) and Canadian citizens.

Cases were all patients newly diagnosed with primary histologically confirmed PCa, actively ascertained through pathology departments across seven French hospitals in the Montreal area between 2005 and 2009. This covered over 80 % of all PCa cases diagnosed in the region of Montreal during the study period according to registry information. Concurrent to case recruitment, controls were randomly selected from the electoral list of French-speaking men residing in the same districts as cases and frequency-matched to cases in 5-year age groups.

Study participants represented 79.4 % of eligible cases and 55.5 % of eligible controls. This study was approved by the Ethics Committees of the following institutions: Institut national de la recherche scientifique, Centre de Recherche du Centre Hospitalier de l’Université de Montréal, Hôpital Maisonneuve-Rosemont, Hôpital Jean-Talon, Hôpital Fleury, and Hôpital Charles-LeMoyne. All participants provided written informed consent.

### Data collection

MetS diagnosis was assigned in a similar fashion among cases and controls, based on the information from the questionnaire. During face-to-face interviews, subjects provided the following information: socio-demographic and anthropometric characteristics (including height and weight at different ages), family history of cancer, and PCa screening history. They were also asked to report any medical condition lasting at least 6 months, including diabetes, hypertension and benign prostate hyperplasia (BPH), and medications taken to treat them, with ages at beginning and end, and duration. Information was collected about lifestyle factors such as physical activity at home, work and leisure, smoking habits, alcohol consumption and dietary habits. Hip and waist circumferences (WC) were measured by the interviewer. The degree of aggressiveness of PCa, defined by the Gleason score, was extracted from prostate biopsy pathology reports and the pre-biopsy prostate specific antigen (PSA) level at diagnosis from patient files.

### MetS definition

We assessed the presence of a MetS history according to definitions from three organizations: the Adult Treatment Panel III from the National Cholesterol Education Program (NCEP-ATPIII) revised by the American Heart Association / National Heart, Lung and Blood Institute, the World Health Organization (WHO) and the International Diabetes Federation (IDF) [6] (Table 1). The prevalence of MetS components (diabetes, hypertension, dyslipidemia and abdominal obesity) was estimated at 2 years before the index date (diagnosis for cases/interview for controls) or at interview for WC-based obesity, and at different ages (20, 40, 50 and 60 years). The timing of MetS onset was based on the first concomitant presence of three individual components. Overall, 1.1 % of cases and 0.8 % of controls whose information about metabolic disorders was not sufficient to conclude about MetS presence were excluded from analyses.

**Table 1:** Criteria, case–control distribution and association between MetS and PCa, according to different MetS definitions

MetS Definition	Criteria used in the study	Cases (*N* = 1937)		Controls (*N* = 1995)		OR^a^	CI 95 %
		*N*	%	*n*	%		
NCEP-ATPIII	At least 3 of these conditions:	476	24.9	629	31.8	0.70	0.60-0.82
	- Dyslipidemia (counting for 2 conditions: low HDL-C and high TG)						
	- Type 2 diabetes						
	- Hypertension						
	- WC > 102 cm (or BMI > 30)						
NCEP-ATPIII ico	NCEP-ATPIII criteria with waist-to-height ratio > 0.5 instead of WC > 102 cm	579	30.3	734	37.2	0.71	0.61-0.82
WHO	Type 2 diabetes + 2 other conditions among them:	149	7.8	279	14.1	0.54	0.44-0.68
	- Hypertension						
	- BMI > 30 or waist-to-hip ratio > 0.9						
	- Dyslipidemia (as a simple condition)						
IDF	- WC > 90 cm for Asiatic men and > 94 cm for others (or BMI > 30) + 2 other conditions among them:	427	22.3	527	26.6	0.75	0.64-0.88
	- Type 2 diabetes						
	- Hypertension						
	- Dyslipidemia (counting for 2 conditions: low HDL-C and high TG)						

^a^Adjusted for age, family history of prostate cancer, ancestry, prostate cancer screening and family income.BMI, Body mass index; CI, Confidence interval; HDL-C, High-density lipoprotein cholesterol; IDF, International Diabetes Federation; NCEP-ATPIII, Adult Treatment Panel III from the National Cholesterol Education Program; OR, Odds ratio; TG: Triglycerides; WC, Waist circumference; WHO: World Health Organization

Blood pressure, fasting glucose, TG and HDL-C blood levels were not available from the study. We used therefore medical histories of hypertension, diabetes and dyslipidemia (hypercholesterolemia, hypertriglyceridemia, or intake of the following lipid lowering drugs: statins, niacin, fibrates, resins or ezetimib). Diabetic subjects receiving insulin therapy since diabetes onset, who never took oral anti-diabetic drugs, were considered not to have T2D. Central obesity was defined on the basis of BMI or WC according to the MetS definitions considered (Table 1). As the appropriate WC cutoff for abdominal obesity may vary by ethnic origin, we also used the waist-to-height ratio (i.e., the Index of Central Obesity: ICO) which has been shown to be more effective in assessing abdominal obesity across ethnicities with a simple cutoff [28]. In sensitivity analyses, we used the NCEP-ATPIII MetS definition which corresponds to the most recent harmonized definition and includes the WC cutoff of 102 cm for abdominal obesity recommended by Health Canada [7].

### Statistical analyses

Unconditional logistic regression was used to determine the risk of PCa associated with MetS. We also assessed the risk of PCa according to age at MetS first onset (≤40, 41–50, 51–60 and > 60 yrs), and according to the number of MetS components.

Risks of low-grade (Gleason scores <7 or 3 + 4) and high-grade (Gleason scores >7 or 4 + 3) PCa [29] were estimated in polynomial logistic models, and their respective regression coefficients were compared using a Wald test. All regression models were systematically adjusted for age (continuous), ancestry (European/Sub-Saharan African/Asian/Greater Middle East/Other/Don’t know), first-degree family history of PCa (Yes/No/Do not know) and recent PSA or Digital Rectal Examination (DRE) screening (No/≤2 years/>2 years/Do not know). The other covariates tested were family income (<$C30 000/$C30 000–79 999/$C80 000 and more/Preferred not to respond/Do not know), education (Primary or secondary/College or university), BPH (Yes/No), ever use of aspirin or 5-alpha-reductase inhibitors (Yes/No), smoking (cigarette pack-years), alcohol consumption (drink-years), physical activity (Very/Moderately/Not very active), dietary habits (annual frequency of fruits and vegetables intake), and changes in fat or sweets intake in the last 20 years (More/Less/No change). Variables retained in the final regression model were those which, when excluded, increased the Akaike Information Criterion by at least 5.

### Sensitivity analyses

The association between MetS and PCa was examined separately in two age groups (<65 vs. ≥65 year-old at index date) to investigate the potential competing risk represented by cardiovascular causes of death, which would be expected to be less common in the younger age group.

An analysis was performed restricting subjects to those screened for PCa (PSA or DRE) within two years of the index date, thereby limiting the inclusion of controls with a potentially undiagnosed PCa. We also ran an analysis restricted to subjects screened with DRE in the last five years, to evaluate the impact on our results of a potentially lower sensitivity of PSA screening due to decreased PSA levels among MetS subjects [30, 31].

We investigated the contribution of T2D in the PCa risk associated with MetS. We examined whether the association between PCa and MetS was different among subjects of Sub-Saharan ancestry.

The risk associated with each component was estimated in a multivariate model including other components. Finally, we assessed whether changes in ORs associated with diabetes and dyslipidemia occurred after adding metformin and statin use (yes/no) in the model.

All analyses were performed using SAS software (9.3; SAS Institute Inc., Cary, NC, USA). A two-sided P value less than 0.05 was considered statistically significant.

## Results

The study population comprised 1937 cases (including 532 high-grade PCa) and 1995 controls. For 3.1 % of cases and 3.9 % of controls interviews were conducted with a proxy, usually the spouse.

Cases were slightly younger than controls (Table 2, p < 0.01). As expected, cases were more likely than controls to have a family history of PCa (p < 0.01), to be of Sub-Saharan ancestry (p < 0.01) and to have been screened for PCa in the last two years (p < 0.01). They were less likely to be of Greater Middle East (p < 0.01) or Asian ancestry (p < 0.01). A regular PCa screening (≥ 5 tests during the previous five years) was more often reported by low-grade than by high-grade cases (59.2 % vs 50.4 %, respectively, p < 0.01). Cases and controls were similar in terms of education, fruit and vegetable consumption, smoking habits and alcohol consumption. Cases had been more physically active than controls during adulthood (p_trend_ = 0.06). Cases had a slightly lower BMI than controls (mean of 26.8 vs 27.2 kg/m^2^, p < 0.01), but had a similar waist circumference (98.6 vs 98.5 cm). Dyslipidemia (29.7 % among cases vs 36.4 % among controls, p < 0.01), hypertension (37.9 % vs 42.3 %, p < 0.01) and T2D (10.6 % vs 17.4 %, p < 0.01) were less frequent among cases, especially when diabetes was diagnosed more than four years before the index date or treated with metformin. Statins and 5-alpha reductase inhibitors uses were similar among cases and controls, whereas aspirin use was more frequent among controls (p = 0.04).

**Table 2:** Characteristics of the PROtEuS study population, Montreal, Canada, 2005-2011

Characteristics	Cases (*N* = 1937)		Controls (*N* = 1995)	
	*n*	%	*n*	%
Age at index date				
<65 years	1009	52.1	896	44.9
≥65 years	928	47.9	1099	55.1
Ancestry				
European	1696	87.6	1686	84.6
Sub-Saharan	130	6.7	90	4.5
Asian	24	1.2	73	3.7
Greater Middle East	45	2.3	99	5.0
Other (Hispanics, Autochtones)	29	1.5	31	1.6
Do not know	12	0.3	14	0.7
Last prostate cancer screening (PSA and/or DRE)				
No	3	0.2	191	9.6
≤2 yrs before index date	1917	99.0	1511	75.7
>2 yrs before index date	1	0.1	235	11.8
Do not know	16	0.8	58	2.9
First-degree family history of prostate cancer				
No	1419	73.3	1739	87.2
Yes	452	23.3	199	10.0
Don't know	66	3.4	57	2.9
Annual family income				
<$C10 000–29 999	490	25.3	497	25.0
$C30 000–79 999	874	45.1	872	43.8
$C80 000- > $C100 000	426	22.0	428	21.5
Preferred not to respond^a^	132	6.8	186	9.3
Do not know	15	0.8	9	0.4
Education				
Primary	449	23.2	429	21.5
Secondary/College	891	46.1	953	47.8
University	592	30.6	611	30.7
Physical activity^b^				
Not very active	444	22.9	503	25.2
Moderately active	518	26.8	545	27.3
Very active	974	50.3	946	47.4
Daily frequency of fruits and vegetables consumption^c^				
≤6	477	24.8	498	25.0
]6-9]	507	26.3	497	25.0
]9-12]	431	22.4	497	25.0
>12	511	26.5	498	25.0
Smoking (pack-years), mean ± SD	22.3	±27.1	23.6	±27.3
Alcohol intake (drink-years), mean ± SD	75.2	±121.6	73.8	±136.6
Body Mass Index 2 yrs ago (kg/m^2^), mean ± SD	26.8	±4.0	27.2	±4.4
Waist circumference (cm), mean ± SD	98.6	±13.6	98.5	±14.3
History of dyslipidemia^c^	576	29.7	727	36.4
Ever use of statin	337	17.4	358	17.9
History of hypertension^c^	732	37.9	842	42.3
Type 2 diabetes^c^	205	10.6	348	17.4
Diagnosed ≥5 years ago	140	7.2	246	12.3
Ever use of Metformin	102	5.3	201	10.1
History of benign prostate hyperplasia^c^	510	26.3	411	20.6
Ever use of Aspirin^c^	308	15.9	367	18.4
Ever use of 5α-reductase inhibitors^c^	33	1.7	46	2.3

^a^Subjects who preferred not to answer were more often from the Middle East and had a lowest educational level than others

^b^Taking into account the reported level of physical activity at home and at work, and the lifetime frequency of leisure activities

^c^Two years before diagnosis / interview

DRE, Digital rectal examination; PSA, Prostate specific antigen; SD, Standard deviation

Overall, 28.4 % of subjects (24.9 % of cases, 31.8 % of controls) ever met MetS criteria according to the NCEP-ATPIII definition (33.8 % if considering the waist-to-height ratio), 11.0 % according to the WHO definition and 24.5 % according to the IDF definition (Table 1). Most subjects with MetS as defined by NCEP-ATPIII had a history of dyslipidemia (94.1 %), hypertension (80.7 %) and/or abdominal obesity (61.0 %), and 35.0 % had a T2D. The MetS profile was different among subjects of Sub-Saharan ancestry, with a higher proportion of T2D (53.9 %) and a lower proportion of dyslipidemia (71.8 %). Among controls, screening in the last two years was more frequent in subjects with MetS than in subjects without MetS (85.9 % vs 74.4 %, p < 0.01), while a history of prostate biopsy was reported in similar proportions (9.4 % vs 8.0 %, p = 0.32). Among cases, median PSA levels did not differ according to the presence or absence of MetS at diagnosis (MetS: 6.0 ng/mL, no MetS: 5.8 ng/mL, p_Wilcoxon_ = 0.12).

After adjustment for age, family history of PCa, ancestry, PCa screening and family income, subjects with a history of MetS (≥3 components according to the NCEP-ATPIII definition) were at significantly lower risk of PCa (OR = 0.70 [0.60-0.82]) as compared to subjects with fewer than three MetS components. The ORs did not vary significantly according to PCa aggressiveness (low-grade: OR = 0.69 [0.58-0.82], high-grade: OR = 0.75 [0.60-0.94]).

In a multivariate model including all the components of MetS together and the same controlling factors as previously, a history of abdominal obesity (OR = 1.09 [0.94-1.27]) or hypertension (OR = 0.93 [0.79-1.08]) were not associated with PCa, but subjects with a history of type 2 diabetes (OR = 0.66 [0.53-0.81]) or dyslipidemia (OR = 0.74 [0.63- 0.86]) were still at decreased risk of PCa. The negative association observed between dyslipidemia and PCa was stronger when adding statins use in the model (OR = 0.58 [0.47-0.71]). Once adjusted on metformin use, the risk associated with T2D was reduced, although no longer significantly (OR = 0.78 [0.59-1.05]).

**Fig. 1:**
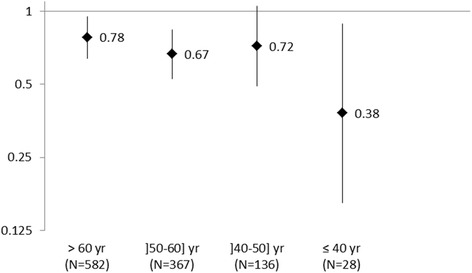
Odds ratio^a^ for the risk of prostate cancer according to age at metabolic syndrome^b^ onset. ^a^Adjusted for age, family history of prostate cancer, ancestry, prostate cancer screening and family income. ^b^According to the definition of the Adult Treatment Panel III from the National Cholesterol Education Program with body mass index instead of waist circumference which was only measured at interview

**Fig. 2:**
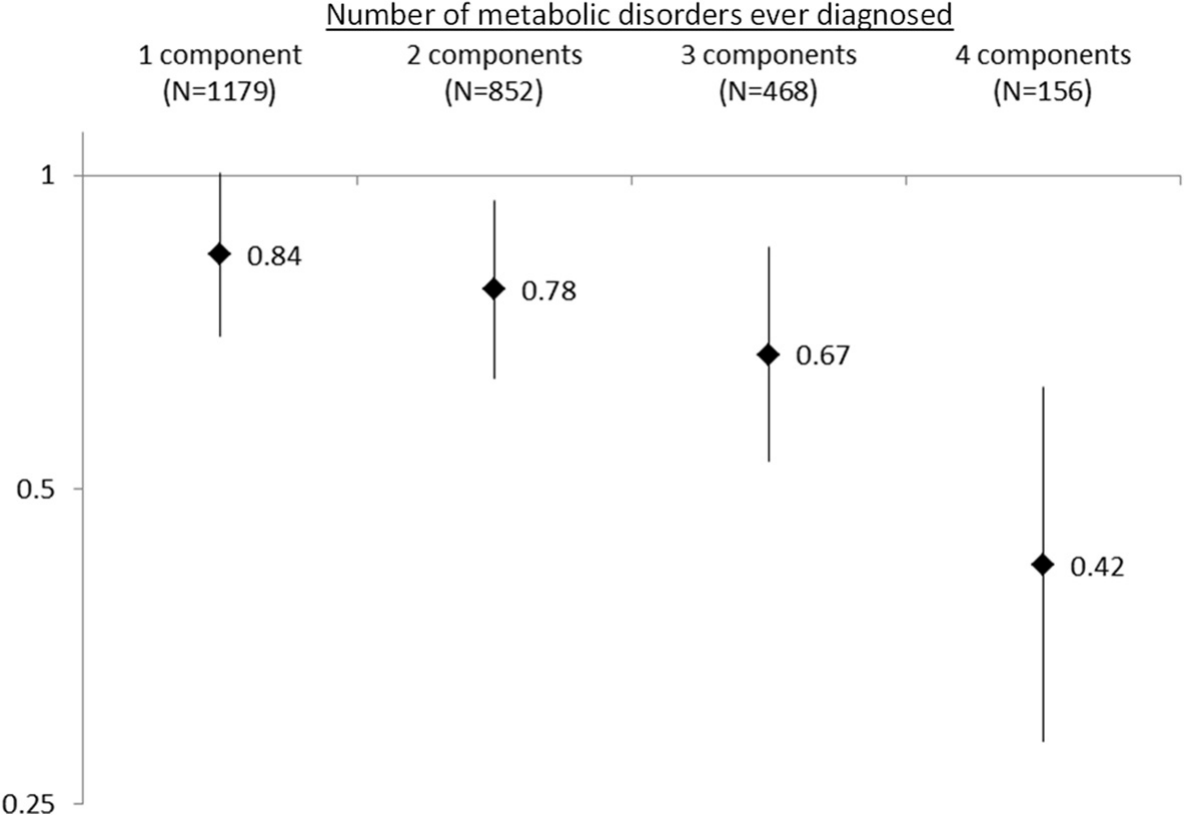
Odds ratio^a^ for the risk of prostate cancer according to number of metabolic syndrome components. ^a^Adjusted for age, family history of prostate cancer, ancestry, prostate cancer screening and family income. The referent category was represented by subjects without any metabolic disorder

The statistically inverse association between a history of MetS and PCa was also observed when using other definitions for MetS or abdominal obesity, with ORs ranging from 0.54 [0.44-0.68] (WHO) to 0.75 [0.64-0.88] (IDF) (Table 1). The negative association tended to be more pronounced among men younger than 40 years at MetS onset (Fig. 1) and among men diagnosed with PCa before age 65 (Table 3). Odds ratios were similar when considering a history of MetS or MetS prevalence at a given time (or age) (data not shown). Using subjects with no MetS component as the referent category did not change the results (data not shown). The risk decreased with the number of MetS components present (Fig. 2, p trend <0.01). This risk decrease was not linear, suggesting rather a synergistic interaction of MetS components under a multiplicative model.

**Table 3:** Association between metabolic syndrome^a^ and prostate cancer risk according to screening, age, ancestry and diabetes

Strata characteristics	N	OR^b^	95 % CI
DRE screened in the last five years	3202		
All cases		0.70	0.60-0.82
Non aggressive cases		0.68	0.57-0.81
Aggressive cases		0.75	0.60-0.95
Screened during the last two years	3387	0.62	0.50-0.78
Age at diagnosis (cases) / interview (controls)			
< 65 years	1892	0.60	0.47-0.76
≥ 65 years	1986	0.79	0.64-0.96
Ancestry			
Sub-Saharan	217	0.80	0.36-1.78
Other (including European, Greater Middle East, Asiatic and Latino ancestries)	3650	0.70	0.60-0.82
Type 2 diabetes			
No	3340	0.80	0.67-0.96
Yes	551	0.67	0.44-1.01

^a^According to the definition of the Adult Treatment Panel III from the National Cholesterol Education Program

^b^Adjusted for age, family history of prostate cancer, ancestry, prostate cancer screening 2 years earlier, and family income

CI, Confidence interval; DRE, Digital rectal examination; OR, Odds ratio

While BPH was positively associated with MetS and PCa, adjusting for BPH did not change the OR associated with MetS. Similar results were observed after exclusion of subjects with T2D, of subjects not screened for PCa in the last two years, or never screened with DRE in the last five years (Table 3). Odds ratios were of the same magnitude among the 217 subjects of Sub-Saharan ancestry as compared to others.

## Discussion

In this population-based case–control study including some 4,000 subjects, we observed a significant inverse relationship between MetS and PCa, regardless of the criteria used to define MetS. This negative association did not vary according to PCa aggressiveness, and was particularly pronounced when MetS was developed at a young age (≤40 years). The analysis of the risk according to the number of MetS components suggests a dose–response relationship with MetS severity.

### Selection bias

Response rates could have affected results if socioeconomic characteristics associated with MetS influenced subjects’ participation. However, according to Canadian census tract data for 2006, the rates for recent immigration, unemployment, low educational level and low household income were similar in living areas of participants and non-participants, both among cases and controls, indicating that selection bias is not of major concern in the study.

### Detection bias

Misclassification of PCa status due to under-detection is possible in both prospective and case–control studies. However, the present study is set in a population with very high PCa screening rates, thereby minimizing chances of a detection bias. Indeed, as a result of a universal access to health care in Montreal, study participants were relatively uniformly and regularly screened for PCa, whatever their socioeconomic position. However controls with MetS were more likely to have been screened recently, which may reflect a closer medical follow-up related to their condition. Nevertheless, this would have increased the probability of PCa diagnosis among MetS subjects, leading to underestimate a true negative association. Furthermore, the similar results observed in analysis restricted to subjects recently screened do not support an important impact of screening frequency on our findings.

Subjects with MetS are known to have decreased PSA levels [30], which may result in a PCa diagnosis at a higher stage and/or grade. This could lead to differential misclassification and overestimation of a negative association between MetS and PCa, especially regarding localized and/or low-grade cases. However, our results did not change substantially when MetS risk was stratified according to PCa grade. Moreover, the association was of the same magnitude among subjects recently screened with DRE, which was shown to improve the predictive value of PCa screening among obese men [32]. Finally, controls with MetS were not less likely to have been referred for prostate biopsy compared to controls without MetS, and PSA levels among cases did not vary significantly according to the presence of MetS at diagnosis. Taken together, these observations argue against an important detection bias due to a decreased sensibility of PSA screening among subjects with MetS.

### MetS exposure classification

A recall bias could be suspected in view of the study design, which would have led to underestimate a negative association. We were able to check the reliability of self-reported MetS-related conditions by referring to patient files in the hospitals where PCas were diagnosed. For respectively 84 %, 94 % and 93 % of cases reporting dyslipidemia, diabetes or hypertension at diagnosis, these disorders were also mentioned in patients’ files, indicating that ascertainment of individual MetS components was reasonably valid. With regard to controls, there is little reason to suspect them of having over-reported MetS-related conditions, thereby driving results towards a protective association. Furthermore subjects and interviewers were blinded to the hypothesis under study and metabolic disorders were not the primary focus of the questionnaire.

Waist circumferences were measured by trained interviewers. While advanced stage PCa can lead to weight loss, only negligible weight losses were recorded in the two years preceding index dates. Moreover in contemporary newly diagnosed PCa, cancer-related weight loss is virtually unseen within two years of diagnosis. When WC was missing, we used the standard threshold for obesity, i.e., a BMI of 30, rather than a BMI value that better corresponds to a WC of 102 cm in our data, to facilitate comparison with previous studies.

Using NCEP-ATPIII criteria, we observed a MetS prevalence of 22 % and 34 % among controls aged 40–59 and ≥60 years, respectively. This prevalence was similar in the younger group but about 20 % lower in the older one in comparison with that reported in two Canadian surveys based on clinical values for blood pressure, TGs, HDL-C and blood glucose [33, 34]. This may reflect a better participation of healthy subjects with limited cardiovascular risk factors in this age group. Indeed, MetS is an indicator for cardiovascular disease risk [35], and competing risk of death from cardiovascular causes was found to bias the association towards negative values [36, 37] or the null value [38] in cohort studies, and may bias the association towards the null value in case–control studies. According to this, our conclusion for a negative association was likely in the correct direction, albeit conservative in terms of magnitude. Besides, the negative association remained significant, and more pronounced with a young age at MetS onset, among subjects aged 65 years and more.

As documented in other investigations [20], T2D was less frequent among PCa patients. However, inclusion of this condition in the MetS definition does not solely explain the inverse relationship observed. The prevalence of diabetes was low compared to other components (35 % of the subjects with MetS) and the negative association with MetS was still significant after adjustment for diabetes or exclusion of diabetic subjects.

About half of subjects with a history of dyslipidemia ever took statins. Statins use has been shown to be associated with a reduced risk of PCa, especially advanced ones [39]. However, statin intake did not contribute to the negative association observed with dyslipidemia in our sensitivity analysis.

### Confounding/effect modification

We were able to consider a wide range of potential confounders. Lifestyle components, including physical activity and diet, did not emerge as important confounding factors in our study population. Subjects of sub-Saharan ancestry were found to have a distinct MetS profile, as observed previously in an American population [40]. However the risk of PCa associated with MetS was similar across ancestries.

### Comparison with previous studies

The literature on the role of MetS in PCa development is divided. No association was found between MetS and PCa risk in two recent meta-analyses, one based on 14 studies (RR = 1.12 [0.93-1.35]) [22] and another based on nine cohort studies (RR = 0.96 [0.85-1.09]) [23]. However, there was a trend for a positive relation between MetS and PCa aggressiveness among PCa patients in another meta-analysis including seven studies (high grade PCa: OR = 1.36 [0.90-2.06], advanced PCa: OR = 1.37 [1.12-1.68]) [23]

The conflicting findings across studies may relate to differences in PCa detection practices between populations. Positive associations were more often observed in Europe [41–44], whereas negative associations, such as ours, were found in North America [45, 46], except among African Americans [40, 47]. This trend might be explained by a less frequent systematic PCa screening in Europe [48], where studies have observed a globally more aggressive cancer profile. Supporting evidence comes from a recent Swedish prospective cohort, using a composite score combining z scores of MetS components [49]. No association was observed with overall risk of PCa, while a positive association emerged for PCa mortality. However, in analyses restricted to cases diagnosed since 1997, the MetS score was significantly associated with a decreased PCa risk. This period was characterized by an increase in low-grade PCa incidence due to more frequent PSA testing. In a more recent analysis taking into account competing events, the decrease in PCa risk observed among men with metabolic disorders was more pronounced in the PSA era [38].

Even when stratified on PCa aggressiveness, results observed in highly screened populations cannot be interpreted in the same way as in other populations. The PCa grade captured at the time of diagnosis depends on screening practices and does not take into account the whole history of PCa progression. For instance, the observed association between MetS and aggressive PCa [42, 50, 51] may ensue from delayed detection among subjects with MetS. Besides, non-screened controls may include non-detected cases, directly impacting case ascertainment.

Other reasons may explain discrepant findings across studies. Two studies and a multicenter clinical trial have been conducted on Spanish [51], Canadian [52] and worldwide [53] patients referred for prostate biopsy. They observed an increased risk of high-grade PCa with MetS, but either no association or a positive one was found with overall PCa risk. In the three studies, PSA levels were elevated among participants [51–53], and the prevalence of MetS was low in the clinical trial [53]. It is thus unclear how findings based on biopsy series compare to those from population-based studies, since exclusion of asymptomatic and untested subjects from control series may have compromised representativeness to the population base, including with respect to MetS prevalence.

Previous investigations have applied different MetS definitions. Interestingly, the four studies that used the NCEP-ATP III MetS criteria with a WC of 102 cm for abdominal obesity and that considered subjects with less than three MetS components as the reference group [46, 50, 51, 54] observed, like us, an inverse association between MetS and PCa risk. While using different MetS definitions did not substantially alter our own findings, it may not be so when applying different criteria to populations presenting diverse anthropometric or clinical patterns.

Finally, methodological issues may be at play. Most previous studies were based on limited numbers of exposed cases. Our study was especially well-powered with some 2000 cases, almost 500 of whom having MetS. Three other recent powerful studies, with more than 300 exposed cases, were recently published on this topic, but these did not consider potential confounding by socioeconomic status, medical history (comorbidities, medication) and/or lifestyle [49, 51, 52]. In another study using a questionnaire-based MetS assessment similar to ours, and reporting a positive association especially in low-grade cases [43], selection (or classification) biases can be suspected in view of the very low MetS prevalence and of socioeconomic differences between cases and hospitalized controls.

One salient advantage of this study is its ability to investigate the role of age at MetS onset in PCa risk which, to our knowledge, has never been investigated. Using a retrospective approach, we could trace the MetS history all along the potential period of PCa development. About half of our subjects with MetS had developed it after age 60. Prospective cohorts have typically relied on a single MetS assessment at baseline including participants of different ages [36, 42, 45, 49, 52, 54, 55], or possibly too young to capture an eventual occurrence of MetS during adulthood [41, 42]. We observed a stronger risk decrease with a young age at MetS onset. In a cohort of men aged 40–49 years, MetS defined using NCEP values was not predictive of PCa [41]. However diabetics, which account for 18 % of our subjects with prevalent MetS at age 40, were excluded from this former cohort.

### Potential mechanisms

Biological pathways involving low insulin, IGF-1 and testosterone levels have been suggested to explain a negative association between MetS and PCa [13]. The pronounced negative association observed with a young age at MetS onset may relate to the timing of diabetes occurrence. Indeed, a reduced risk of PCa is usually observed only several years after diabetes diagnosis [56], probably because long-standing diabetics may experience low insulin levels in later years. Besides, the apparent contribution of dyslipidemia to the negative association observed raises the issue of the role of cholesterol in prostate malignancy, although some evidence suggests that it might be positively related with PCa growth [57–60]. Conversely, a cholesterol-lowering effect of cancer has been suggested, as a result of tumor metabolism. Low cholesterol-cancer associations have been mostly observed in studies conducted before introduction of PSA-testing, including more advanced PCas, or in PSA-screened populations where cholesterol was measured within one year of cancer diagnosis [61]. The high PSA-screening rate in our population and our application of a two-year-lag in the analysis between MetS assessment and PCa diagnosis do not support such a reverse relation. Future experimental research exploring potential biological mechanisms of MetS should consider the synergistic interaction of MetS components.

## Conclusion

Our findings provide evidence for a negative association between a history of MetS and PCa risk in a population regularly screened for PCa.

These findings can be interpreted in two ways. First, while it likely had a marginal influence on our results, under-detection of PCa among MetS patients remains possible. This issue should be addressed in future epidemiological studies. Second, a synergistic interaction between metabolic factors can be at play, with diabetes and dyslipidemia as main actors.

Large studies including repeated biological measurements over time are required to confirm the role of timing at MetS onset on PCa development.

## Abbreviations

BMI: (Body mass index)
CI: (Confidence interval)
DRE: (Digital rectal examination)
HDL-C: (High-density lipoprotein cholesterol)
IDF: (International Diabetes Federation)
MetS: (Metabolic syndrome)
NCEP-ATPIII: (Adult Treatment Panel III from the National Cholesterol Education Program)
OR: (Odds ratio)
PCa: (Prostate cancer)
PSA: (Prostate specific antigen)
TG: (High triglycerides)
WC: (Waist circumference)
WHO: (World Health Organization)
